# In Silico Design and Immunogenicity Evaluation of a Multiepitope Vaccine Candidate Against *Leishmania tropica* in BALB/c Mice

**DOI:** 10.1155/japr/3953857

**Published:** 2026-10-19

**Authors:** Sara Idriss, Almoutassem Billah Zetoune, Chadi Soukkarieh

**Affiliations:** ^1^ Department of Biochemistry and Microbiology Faculty of Pharmacy Damascus University Damascus Syria damascusuniversity.edu.sy; ^2^ Department of Animal Biology Faculty of Science Damascus University Damascus Syria damascusuniversity.edu.sy

**Keywords:** BALB/c mice, cutaneous leishmaniasis, *Leishmania tropica*, multiepitope vaccine, recombinant protein, Th1 response

## Abstract

**Background:**

Cutaneous leishmaniasis caused by *Leishmania tropica* remains a major neglected parasitic disease with limited therapeutic options and frequent treatment failure, highlighting the urgent need for effective vaccine‐based interventions. Multiepitope vaccine (MEV) strategies provide a rational and targeted approach to induce defined cellular and humoral immune responses against *Leishmania* infection.

**Methods:**

In this study, a MEV candidate was designed using conserved cytotoxic T lymphocyte (CTL), helper T lymphocyte (HTL), and linear B‐cell epitopes derived from immunodominant *Leishmania* antigens. Prior to experimental evaluation, protein–protein docking was performed to assess the interaction between the vaccine candidate and the TLR4 receptor. Molecular dynamics (MD) simulations were subsequently conducted using NAMD 3.0.0 with the CHARMM36 force field under physiological conditions (TIP3P water model, 0.15 M NaCl, pH 7.4). Structural parameters including root mean square deviation (RMSD), radius of gyration (Rg), and principal component analysis (PCA) were calculated. The recombinant construct was expressed in an *Escherichia coli* pET‐20b (+) system and purified using affinity chromatography. The vaccine was evaluated in a BALB/c mouse model using a multidose subcutaneous immunization protocol followed by *L. tropica* challenge. Immunological responses were assessed by quantitative real‐time PCR analysis of cytokine gene expression (IL‐12, IFN‐*γ*, and IL‐4) and by Western blot analysis for detection of antigen‐specific antibodies. Safety was evaluated based on survival and observation of apparent adverse effects during the experimental period.

**Results:**

Computational analysis demonstrated stable interaction between the vaccine candidate and the TLR4 receptor. MD simulations confirmed the formation of a stable and compact vaccine–TLR4 complex, supported by RMSD, Rg, and PCA analyses, indicating reduced conformational fluctuations and enhanced receptor stabilization compared with the unbound state. Immunological analysis revealed a strong Th1‐skewed immune response characterized by significant upregulation of IFN‐*γ*, modulation of IL‐12 expression, and marked downregulation of IL‐4 following challenge. Western blot analysis confirmed specific antigen recognition in vaccinated mice, whereas control groups showed no detectable reactivity. No mortality, behavioral changes, or apparent vaccine‐related adverse effects were observed during the observation period, supporting the tolerability of the vaccine under the experimental conditions.

**Conclusion:**

Overall, the MEV induced coordinated cellular immune responses together with antigen‐specific antibody recognition. The observed cytokine gene expression profile and antibody reactivity indicate a robust immunogenic response and support the recombinant construct as a promising candidate for further preclinical evaluation against cutaneous leishmaniasis. Additional studies assessing protective efficacy, parasite burden, and long‐term immune durability are warranted.

## 1. Introduction

A recurring global health concern, leishmaniasis is caused by the protozoan parasite *Leishmania* and is transmitted by infected phlebotomine sandflies [1–3]. Cutaneous leishmaniasis (CL) is the most prevalent clinical form of the disease and is characterized by nodular lesions, chronic ulcers, and various cutaneous manifestations. The main causative species include *Leishmania major* (*L. major*) and *Leishmania tropica* (*L. tropica*) [1–3]. *L. major* is predominantly distributed across the Near and Middle East, Central Asia, and West, North, and East Africa, whereas *L. tropica* is mainly associated with urban regions of the Near and Middle East and northern Africa, where it primarily infects humans [1–4]. Despite the availability of chemotherapeutic agents for CL, their clinical efficacy is limited due to nephrotoxicity, reduced effectiveness, relapse rates, and treatment cost considerations. Accordingly, vaccination is considered one of the most promising strategies for controlling leishmaniasis [5].

Protective immunity against *Leishmania* is largely mediated by the balance between T‐helper 1 (Th1) and T‐helper 2 (Th2) responses. Th1 cells promote protection through the production of interferon gamma (IFN‐*γ*), which activates macrophages and enhances parasite killing, whereas Th2 responses, characterized by interleukin (IL)‐4 secretion, can suppress macrophage activation and favor disease progression. CD8+ T cells also contribute significantly by producing IFN‐*γ* and supporting the differentiation of protective Th1 responses [6]. These cells are particularly important in controlling primary low‐dose infections and preventing reinfection, making them key targets for vaccine design [7]. IgG2a is widely recognized as a Th1‐associated antibody isotype and has been used as an indicator of a Th1‐biased immune response in murine *Leishmania* vaccination studies, although it should not be considered an independent correlate of protection [8]. Despite ongoing efforts, the development of reliable vaccines against CL remains challenging. Traditional approaches based on live attenuated or killed whole‐parasite vaccines have shown limited success in clinical settings, highlighting the need for alternative strategies [9]. In this context, epitope‐based multiepitope vaccines (MEVs) represent a promising approach by focusing on specific immunogenic components rather than whole organisms [10]. However, the immunogenicity of peptide‐ and epitope‐based formulations may require enhancement through appropriate adjuvants. Toll‐like receptor 4 (TLR4)–related innate immune signaling has been implicated in the immune and inflammatory response to *Leishmania*, and has therefore been investigated as a potential immunomodulatory pathway in vaccine‐related strategies [11].

In the present study, immunogenic B‐ and T‐cell epitopes were selected from 10 *Leishmania* proteins representing diverse antigenic sources. These included *Leishmania* elongation initiation factor (LeIF) and *L. major* stress‐inducible protein 1 (LmSTI1), which have been investigated as defined vaccine antigens capable of inducing cellular immune responses [12]; kinetoplastid membrane protein‐11 (KMP11), glycoprotein 63 (gp63), and promastigote surface antigen‐2 (PSA‐2), which have been investigated as candidate *Leishmania* vaccine antigens [13, 14]; and heat shock protein 70 (HSP70) and histone H2A (H2A), which have been incorporated into experimental multiantigen vaccine formulations [15]. Nucleoside hydrolase (NH) was included as an additional antigenic source with documented immunogenic and vaccine‐related properties [16]. P27 and L5 were included as additional *Leishmania* antigenic sources in the multiantigen design; notably, L5 has previously been evaluated as a DNA vaccine candidate against *L. tropica* [17]. The selected B‐ and T‐cell epitopes derived from these proteins were subsequently assembled into a chimeric MEV construct, thereby combining epitopes from multiple parasite antigens rather than relying on a single antigenic source.

To provide an additional immunostimulatory component, the heparin‐binding hemagglutinin adhesin (HBHA), a surface‐associated mycobacterial protein identified in *Mycobacterium tuberculosis*, was incorporated into the construct [18]. HBHA has been reported to possess immunostimulatory properties mediated, at least in part, through TLR4‐dependent activation of antigen‐presenting cells. In particular, HBHA can promote dendritic‐cell maturation and increase the expression of costimulatory molecules, including CD40, CD80, and CD86, as well as MHC Classes I and II molecules. It has also been reported to enhance the production of inflammatory cytokines, including IL‐12, and to promote the activation of T cells with a Th1‐associated cytokine profile [18]. Thus, HBHA was incorporated as a separate immunostimulatory component intended to support innate immune activation and facilitate subsequent antigen presentation and T‐cell responses, whereas the selected *Leishmania* proteins served as the sources of vaccine epitopes. Accordingly, the construct combined epitope sequences derived from multiple *Leishmania* antigens with an immunostimulatory component rather than relying on a single antigenic source. The resulting construct was evaluated computationally for its physicochemical properties, antigenicity, allergenicity, toxicity, structural characteristics, and predicted interaction with the TLR4/MD‐2 complex.

The vaccine candidate was subsequently evaluated experimentally in BALB/c mice to characterize its preliminary immunogenicity. Cytokine gene expression profiles of IL‐12, IFN‐*γ*, and IL‐4 were assessed following administration of the vaccine candidate and after *L. tropica* challenge to characterize the induced immune response. In addition, antigen‐specific antibody responses were examined following challenge. Thus, this study integrates immunoinformatics‐based vaccine design with preliminary in vivo immunogenicity evaluation of a MEV candidate against *L. tropica.*

## 2. Materials and Methods

### 2.1. Ethics Statement

All animal procedures, including immunization, challenge, sample collection, and sacrifice, were performed in accordance with the ethical guidelines and recommendations for the care and use of laboratory animals and were approved by the Animal Ethics Committee of Damascus University, Syria (Approval No. PH‐200825‐485). Mice were sacrificed according to approved humane procedures and institutional guidelines.

### 2.2. Design and Computational Analysis of the MEV

The MEV construct used in this study was designed and computationally evaluated by our group prior to experimental validation. Immunogenic epitopes were selected from conserved and immunodominant proteins of *L. major* and *L. tropica*, based on population‐specific human HLA allele distribution as well as murine MHC alleles (H‐2) to ensure both broad population coverage and experimental compatibility with BALB/c mice.

The overall computational workflow of vaccine design and epitope selection is described in Material S1. The bioinformatic tools, prediction servers, and selection criteria (including threshold values and antigenicity, allergenicity, and toxicity filters) used for epitope prediction and screening are summarized in Table S1. In addition, the population‐specific HLA allele distribution and the complete list of predicted cytotoxic T lymphocyte (CTL) and helper T lymphocyte (HTL) epitopes prior to final screening are provided in Material S2.

CTL and HTL epitopes were derived from NH (Q4QDN1), H2A (A0A142C7A6), HSP70 (Q94594), LmSTI1 (A0A165DNF6), PSA‐2 (Q9NDD1), L5 (A0A5Q2WYE3), P27 (A0A482D292), KMP11 (A0A482KHF1), LeIF (W5XL77), and gp63 (Q8MNZ1), whereas linear B‐cell epitopes were selected from the surface‐exposed proteins gp63 and KMP11. To enhance antigen processing and presentation, CTL, HTL, and B‐cell epitopes were linked using AAY, GPGPG, and KK linkers, respectively, whereas the HEYGAEALERAG linker was inserted between CTL and HTL regions to improve structural separation.

An adjuvant sequence consisting of HBHA from *M. tuberculosis* (UniProt Accession: P9WIP9) was fused to the N‐terminus of the construct and linked to the PADRE sequence (AKFVAAWTLKAAA) through an EAAAK linker. HBHA was incorporated as a protein adjuvant/immunostimulatory component based on its previously reported immunostimulatory properties. A His×6 tag was added at the C‐terminus via an RVRR linker to facilitate purification.

Physicochemical properties of the final construct were analyzed using the ProtParam server (ExPASy). The interaction between the MEV construct and TLR4 was subsequently evaluated by protein–protein docking using the ClusPro 2.0 server. Docked complexes were ranked according to cluster size and binding energy, and the top‐scoring pose was selected for further analysis.

Molecular dynamics (MD) simulation of the selected vaccine–TLR4 complex was then performed using NAMD Version 3.0.0 with the CHARMM36 force field under physiological conditions (TIP3P water model, 0.15 M NaCl, pH 7.4). The stability of the complex throughout the simulation was assessed by analyzing RMSD, RMSF, radius of gyration (Rg), and hydrogen‐bond profiles.

### 2.3. Molecular Cloning, Expression, and Purification of rMEP

The gene encoding the designed multiepitope protein (MEP) was codon‐optimized to enhance its translational efficiency in *Escherichia coli* and subsequently cloned into the pET20b(+) expression vector (GenScript, United States). The recombinant plasmid was introduced into *E. coli* BL21 (DE3) competent cells using the heat‐shock transformation method. Following successful transformation, protein expression was induced by the addition of isopropyl *β*‐D‐1‐thiogalactopyranoside (IPTG) at a final concentration of 0.5 mM. Induction was carried out at 37°C for 4 h, a condition that was determined to be optimal for rMEP expression based on preliminary optimization experiments. Subsequently, large‐scale bacterial cultures were grown under the same optimized conditions to obtain sufficient quantities of the recombinant protein. The expressed recombinant MEP, harboring a C‐terminal 6×His tag, was purified using His‐affinity chromatography, which exploits the specific interaction between histidine residues and immobilized nickel ions. Purification was performed using the Elabscience His Tag (HHHHHH) Fusion Protein Purification Kit in accordance with the manufacturer′s instructions. The purified protein was then collected and prepared for subsequent analyses.

### 2.4. Immunization and Challenge of BALB/c Mice

A total of 24 female BALB/c mice, 4 weeks old and pathogen‐free, were randomly assigned to two groups (*n* = 12 per group). The control group received 200 *μ*L of PBS, whereas the experimental group received three subcutaneous doses, with each dose containing 50 *μ*g of purified MEP in 200 *μ*L PBS, administered at 15‐day intervals.

Two weeks after the third immunization, three mice from each group were sacrificed, and cervical lymph nodes were collected. The challenge parasite consisted of a Syrian strain of *L. tropica* promastigotes (LCEBS, Damascus, Syria), cultured in RPMI 1640 medium (Lonza, Switzerland) supplemented with 5% fetal bovine serum (Cytogen, Germany). Promastigotes in the stationary growth phase were used for challenge. Two weeks after the third immunization, both vaccinated and PBS control groups were challenged by subcutaneous injection of 1 × 10^6^ *L. tropica*promastigotes in 200 *μ*L PBS into the plantar surface of the right hind footpad.

On Day 5 postchallenge, blood samples were collected from three mice per group for antibody detection before they were sacrificed for popliteal lymph node collection. Popliteal lymph nodes were also collected from mice sacrificed at 4 weeks postchallenge. The final three mice per group were maintained for up to 2 months to monitor survival and any visible adverse effects. All experimental procedures, including immunization, challenge, sample collection, and observation, are summarized in Figure 1.

**Figure 1:**
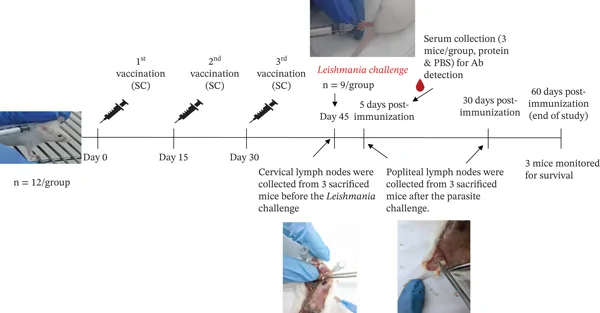
Schedule of immunization and *L. tropica* challenge: Mice were divided into two groups (*n* = 12). The experimental group received three subcutaneous doses of 50 *μ*g MEP, whereas controls received PBS. Cervical lymph nodes were collected 2 weeks after the last immunization. Both the MEP‐immunized group and the PBS control group were subjected to *L. tropica* challenge following the immunization protocol via subcutaneous injection into the plantar surface of the right hind footpad. Popliteal lymph nodes were collected at 5 days and 4 weeks postchallenge. Blood was collected from three mice at Day 5 for antibody detection. The final three mice per group were observed for up to 2 months for survival and visible adverse effects.

### 2.5. Evaluation of Cytokine mRNA Expression in Draining Lymph Nodes by Real‐Time PCR

RNA was isolated from immune cells obtained from cervical and popliteal lymph nodes of mice from both control and experimental groups using the GF‐1 Total RNA Extraction Kit (Vivantis, Malaysia). Complementary DNA (cDNA) was synthesized using Power cDNA Synthesis Kit (iNtRON Biotechnology, Korea) from the extracted RNA and used for quantitative analysis of inflammatory cytokine gene expression by real‐time PCR. The expression levels of IL‐12, IL‐4, and IFN‐*γ* were assessed using a Rotor‐Gene real‐time PCR system and HOT FIREPol EvaGreen qPCR Mix Plus (ROX). PCR amplification was carried out for 35 cycles following an initial denaturation at 95°C for 12 min. Each cycle consisted of denaturation at 95°C for 20 s, primer annealing at 60°C for 20 s, and extension at 72°C for 20 s. DNA amplification was performed by DNA polymerase using free nucleotides present in the reaction mixture. The primer sequences used in this study are listed in Table 1. The primers were synthesized by metabion international AG (Germany).

**Table 1:** Primer sequences used in this study [19–21].

Oligo name	Sequence
GAPDH (FW)	AACTTTGGCATTGTGGAAGG
GAPDH (RE)	GGATGCAGGGATGATGTTCT
IFN‐y (FW)	GCTCTTCCTCATGGCTGTTT
IFN‐y (RE)	GTCACCATCCTTTTGCCAGT
IL‐4 (FW)	CGAAGAACACCACAGAGAGTGAGCT
IL‐4 (RE)	GACTCATTCATGGTGCAGCTTATCG
IL‐12 (FW)	GGAAGCACGGCAGCAGAATA
IL‐12 (RE)	AACTTGAGGGAGAAGTAGGAATGG

### 2.6. Western Blot Analysis

Total *Leishmania* promastigote proteins, along with the purified recombinant multiepitope protein, were separated by SDS–PAGE using polyacrylamide gels under denaturing conditions. Prior to electrophoresis, protein samples were mixed with loading buffer and heat‐denatured at 95°C for 5 min. Approximately 25 *μ*g of total protein per lane was loaded for analysis. Protein separation was performed under constant voltage until adequate resolution of protein bands was achieved. Following electrophoresis, proteins were transferred onto nitrocellulose membranes using a wet transfer system (Bio‐Rad) at 100 mA for 75 min in cold transfer buffer to ensure efficient protein transfer while minimizing heat generation. Transfer efficiency was confirmed using a prestained molecular weight marker. Membranes were blocked overnight at room temperature with blocking buffer to prevent nonspecific binding sites. After blocking, membranes were washed three times with TBS‐T (Tris‐buffered saline containing 0.1% Tween‐20), 10 min per wash, under gentle agitation. Membranes were then incubated for 1 h at room temperature with primary antibodies consisting of individually collected sera from three vaccinated mice and three PBS‐injected control mice, 5 days postchallenge. Sera were diluted at 1:500 (v/v) in blocking buffer and applied under gentle shaking to ensure uniform binding. After incubation, membranes were washed three times with TBS‐T (10 min each wash) to remove unbound antibodies. Subsequently, membranes were incubated for 1 h at room temperature with alkaline phosphatase (AP)‐conjugated goat anti‐mouse IgG secondary antibody diluted 1:1000 (v/v). Following secondary antibody incubation, membranes were washed three times with TBS‐T under the same conditions. Protein bands were developed using NBT/BCIP substrate solution (33 *μ*L nitro‐blue tetrazolium and 33 *μ*L 5‐bromo‐4‐chloro‐3‐indolyl phosphate in 10 mL AP buffer) in the dark with gentle agitation until clear immunoreactive bands appeared. The reaction was stopped by washing the membranes with distilled water.

### 2.7. Statistical Analysis

Gene expression levels were determined from cycle threshold (Ct) values and expressed as relative fold changes using the 2^−*Δ* *Δ* *C* *t*^ method. The PBS‐treated group served as the calibrator (reference control) and was set to 1. Expression levels were normalized to a housekeeping gene (GAPDH). Data normality was assessed using the Shapiro–Wilk test prior to statistical analysis. Differences in cytokine gene expression between the protein‐immunized group and the PBS control group across the studied time points (prechallenge, 5 days, and 30 days postchallenge) were analyzed using one‐way analysis of variance (ANOVA). Post hoc comparisons were performed using the Bonferroni test. A *p* value < 0.05 was considered statistically significant. Cytokine gene expression analysis focused on IL‐12, IL‐4, and IFN‐*γ* to evaluate Th1 and Th2 immune responses. All statistical analyses were performed using IBM SPSS Statistics for Windows, Version 25.0 (IBM Corp., Armonk, New York, United States).

## 3. Results

### 3.1. Vaccine Construct

The designed MEV construct successfully integrated five CTL epitopes, six HTL epitopes, and two linear B‐cell epitopes into a single chimeric sequence. The construct also included an N‐terminal adjuvant (HBHA fused with PADRE) and a C‐terminal His×6 tag to facilitate purification. The complete amino acid sequence of the designed vaccine construct is provided in Material S3. Physicochemical analysis using ProtParam indicated that the construct is stable and basic in nature, with a molecular weight of 47,860.89 Da and favorable stability‐related parameters, including instability index, aliphatic index, and GRAVY score. Protein–protein docking analysis revealed a strong binding affinity between the vaccine construct and the TLR4 receptor, with the best docked model selected based on the lowest binding energy (−967 kcal/mol) among 15 generated complexes (Figure S1).

### 3.2. MD Simulation

To investigate the stability and dynamic behavior of the complex under physiological conditions, a 200‐ns MD simulation was conducted. The RMSD analysis showed that the vaccine–TLR4 complex stabilized after an initial equilibration phase and maintained consistent structural behavior throughout the simulation. In addition, the Rg indicated that the complex preserved a compact structure with moderate flexibility, suggesting a stable interaction within a dynamic environment.

Overall, these results demonstrate that the vaccine–TLR4 complex remains structurally stable under physiological conditions, supporting its potential biological relevance.

Figure 2A illustrates the RMSD profiles of TLR4–MD2 in unbound, lipopolysaccharide (LPS)‐bound, and vaccine‐bound states. The unbound complex exhibited the highest fluctuations, reflecting substantial conformational flexibility and stabilizing only after ~20 ns while maintaining broader variation (4–6 Å). In contrast, the LPS‐bound complex showed rapid stabilization and consistently low RMSD values, indicating increased structural rigidity upon ligand binding. The vaccine‐bound complex displayed intermediate RMSD values (2–4 Å), suggesting a balance between structural stability and flexibility.

**Figure 2:**
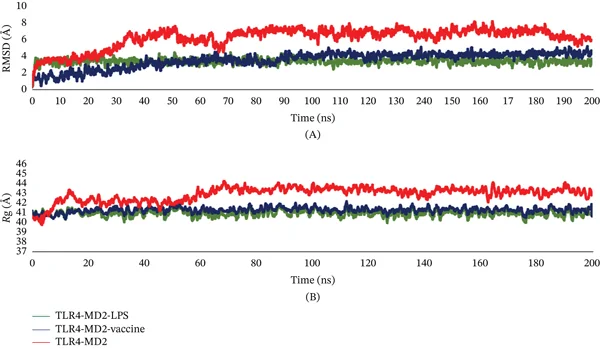
Stability (RMSD) and compactness (Rg) of TLR4‐MD2 complexes over a 200‐ns simulation. (A) Panel A shows the RMSD, indicating structural stability: The LPS‐bound complex (green) is the most stable, followed by the vaccine‐bound complex (blue), with the unbound complex (red) showing the greatest instability. (B) Panel B presents the Rg, reflecting compactness: The LPS‐bound complex is the most compact, the unbound complex is the most expanded, and the vaccine‐bound state maintains an intermediate structure, balancing flexibility and stability.

Figure 2B presents the Rg profiles, reflecting the compactness of the complexes. The unbound system showed the highest Rg values (41–44 Å), indicating an expanded and dynamic conformation. The LPS‐bound complex remained more compact (40–42 Å), consistent with a stable structural state. The vaccine‐bound complex exhibited intermediate compactness, maintaining structural integrity while allowing limited conformational adaptability.

Overall, ligand binding significantly influences the structural stability of TLR4–MD2. Whereas the LPS‐bound state represents the most stable and compact configuration, the vaccine‐bound complex maintains a stable yet flexible conformation that may be beneficial for biological function. In contrast, the unbound complex shows increased structural fluctuation and reduced compactness.

Principal component analysis (PCA) was performed to explore the conformational space and collective motions of the TLR4–MD2 system in unbound, LPS‐bound, and vaccine‐bound states. The unbound complex exhibited high conformational variability, reflecting a broad and flexible dynamic behavior. In contrast, both the LPS‐ and vaccine‐bound systems showed more restricted conformational spaces with substantial overlap between the two states.

As shown in Figure 3A, the unbound complex formed a distinct cluster (red), indicating heterogeneous conformational sampling. Conversely, the vaccine‐bound (blue) and LPS‐bound (green) systems clustered closely and overlapped significantly, suggesting similar stabilization patterns induced by ligand binding.

**Figure 3:**
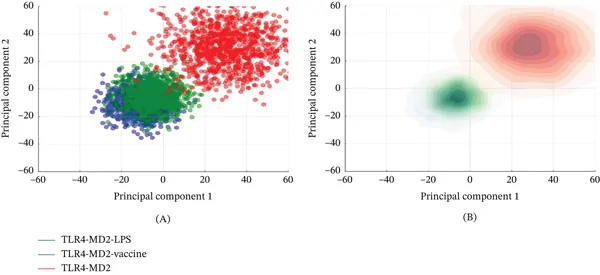
Principal component analysis (PCA) of TLR4‐MD2 complexes. This figure illustrates the PCA of TLR4‐MD2 complexes in three different states: unbound (red), LPS‐bound (green), and vaccine‐bound (blue). (A) Panel A shows the clustering of conformational states in the principal component space, highlighting distinct separation for the unbound complex (red) and significant overlap between the LPS‐bound (green) and vaccine‐bound (blue) complexes. This overlap indicates that both ligands induce a similar stabilizing effect. (B) Panel B presents the density contours, further emphasizing the similarity in the conformational responses of the LPS‐ and vaccine‐bound complexes. Both ligands constrain TLR4‐MD2 into a comparable structural space, suggesting that the vaccine candidate effectively mimics the stabilization induced by LPS.

Density contour analysis (Figure 3B) further supported these observations. The unbound system displayed a widely distributed conformational landscape, whereas both the LPS‐ and vaccine‐bound complexes exhibited dense and overlapping regions, indicating constrained and stable motions. These findings suggest that the vaccine construct induces a conformational behavior in TLR4–MD2 comparable with that of LPS, supporting a similar stabilizing effect on the receptor.

### 3.3. Protein Extraction and Purification

The recombinant multiepitope protein was successfully expressed in *E. coli* using the pET‐20b (+) vector and induced with IPTG. SDS‐PAGE analysis revealed a distinct protein band at approximately 47.86 kDa, which was absent in uninduced cells, confirming successful expression. Optimal expression was achieved at 37°C after 4 h of induction. The His‐tagged protein was subsequently purified using affinity chromatography, and analysis of the eluted fractions showed a prominent band corresponding to the target protein, indicating successful purification with high purity, as illustrated in Figure 4.

**Figure 4:**
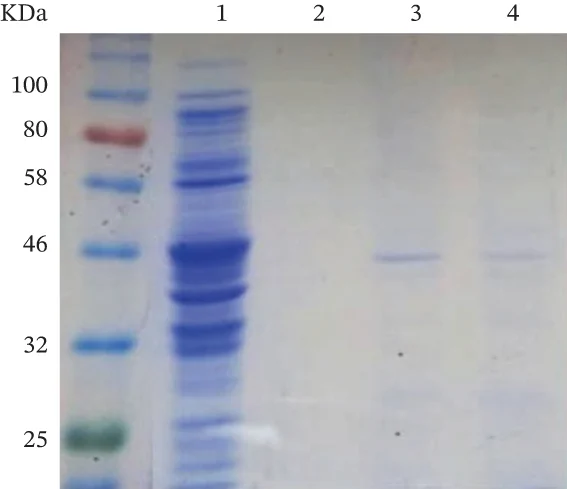
SDS‐PAGE of the His‐tagged protein purification. Lane 1, unbound proteins; Lane 2, final wash; and Lanes 3 and 4, purified eluted protein.

### 3.4. Temporal Changes in Cytokine Gene Expression Following Protein Immunization and Challenge

In the protein‐immunized group, cytokine gene expression showed a Th1‐associated pattern from the prechallenge period through 30 days after *L. tropica* challenge (Figure 5). IL‐12 expression decreased markedly from 252.13 ± 127.00 before challenge to 2.08 ± 0.50 at 5 days postchallenge, followed by a partial increase to 19.49 ± 5.21 at 30 days postchallenge. In contrast, IL‐4 expression progressively decreased from 11.84 ± 7.37 before challenge to 2.00 ± 0.77 at 5 days and 0.002 ± 0.001 at 30 days postchallenge. IFN‐*γ* expression showed an opposite pattern, increasing from 2.27 ± 1.50 before challenge to 6.89 ± 2.33 at 5 days and reaching 39.41 ± 3.88 at 30 days postchallenge (Figure 6). Significant differences were observed among the three time points for IL‐12, IL‐4, and IFN‐*γ* (*p* < 0.05). Overall, the cytokine expression profile from the prechallenge period through 5 and 30 days postchallenge, characterized by increasing IFN‐*γ* and decreasing IL‐4 expression together with the temporal changes in IL‐12, indicates a sustained Th1‐associated immune response following immunization and subsequent *L. tropica* exposure.

**Figure 5:**
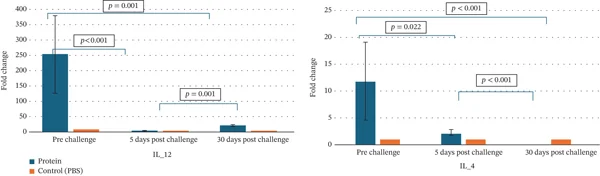
Temporal changes in IL‐12 and IL‐4 gene expression fold change in the protein‐immunized group relative to the PBS control group. Gene expression levels were determined using the 2^−*Δ* *Δ* *C* *t*^ method and normalized to a housekeeping gene. Data are presented as mean ± SD. IL‐12 expression fold change showed a marked decrease at 5 days postchallenge, followed by a partial increase at 30 days postchallenge, whereas IL‐4 expression fold change decreased progressively, reaching its lowest level at 30 days postchallenge. Statistically significant differences between the indicated time points are shown in the figure.

**Figure 6:**
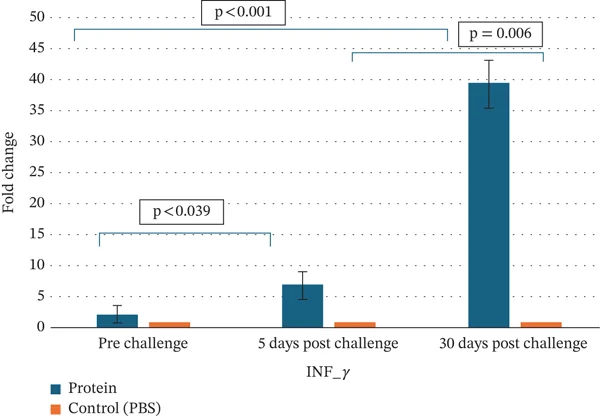
Temporal changes in IFN‐*γ* gene expression fold change in the protein‐immunized group. Expression levels (fold change) were calculated relative to the PBS control group using the 2^−*Δ* *Δ* *C* *t*^ method. Data are presented as mean ± SD. IFN‐*γ* expression increased from prechallenge to 5 days postchallenge and reached its highest level at 30 days postchallenge. Statistically significant differences between the indicated time points are shown in the figure.

### 3.5. Western Blot Detection of Early Antigen‐Specific Antibody Response 5 Days After Challenge With *Leishmania* Protein Vaccine

The membranes were incubated individually with sera collected from three vaccinated mice and three PBS control mice at 5 days postchallenge. Sera from vaccinated mice exhibited clear immunoreactivity against both native *Leishmania* proteins and the recombinant vaccine antigen, indicating the induction of antigen‐specific antibodies. In contrast, sera obtained from the PBS control group showed no detectable immunoreactive bands. Notably, immunodominant bands were observed at approximately 11 and 63 kDa in vaccinated samples, whereas no corresponding reactivity was detected in control samples. For clarity, a representative immunoblot obtained from one vaccinated mouse is shown in the figure. Similar qualitative immunoreactive patterns were observed among the tested vaccinated sera; however, only a representative blot is presented for visualization purposes (Figure 7).

**Figure 7:**
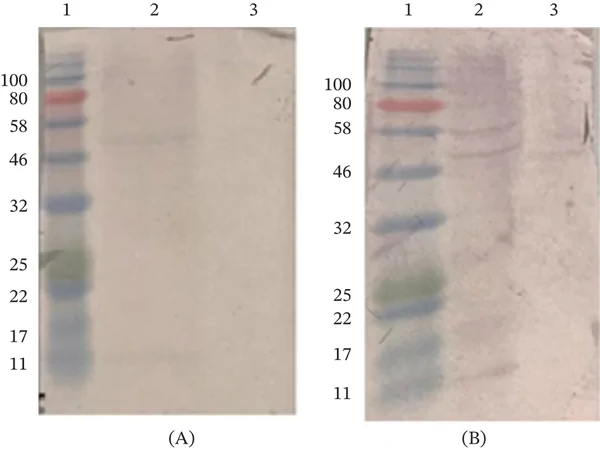
Western blot analysis of antibody responses against *Leishmania* proteins and protein vaccine 5 days postchallenge. (A) PBS‐control sera showed no detectable immunoreactivity. (B) Vaccinated sera showed specific reactivity to both parasite proteins and the vaccine antigen, with dominant bands at ~11 and 63 kDa. Lane 1: molecular weight marker; Lane 2: total *Leishmania* proteins; Lane 3: recombinant vaccine protein.

### 3.6. Survival and Clinical Observation

Three mice from each group were monitored longitudinally from the beginning of immunization throughout the immunization period, during the interval preceding parasite challenge, and following *L. tropica* challenge for up to 2 months. During the postimmunization period prior to challenge, no mortality, abnormal behavior, visible signs of illness, or apparent local or systemic adverse effects associated with vaccination were observed in the vaccinated groups. Similar observations were made in the PBS control group. Following parasite challenge, no mortality or apparent vaccine‐related adverse effects were observed during the subsequent observation period. Overall, no apparent adverse effects attributable to the recombinant MEV were observed under the experimental conditions.

## 4. Discussion

This study describes the design, expression, and immunological evaluation of a MEV candidate against *L. tropica*, the causative agent of CL. The findings indicate that the recombinant construct induced measurable immune responses characterized by a Th1‐associated cytokine profile, including enhanced IFN‐*γ* expression and reduced IL‐4 expression following immunization and challenge. These results support the potential of the designed multiepitope construct as a candidate for further investigation.

The vaccine was rationally designed using conserved and immunodominant *Leishmania*‐derived epitopes to ensure broad immune coverage. The construct included CTL and HTL epitopes derived from multiple *Leishmania* antigens to enhance antigen presentation and T‐cell activation diversity. In parallel, B‐cell linear epitopes derived from GP63 and KMP11 were incorporated to promote antigen‐specific antibody responses against parasite surface antigens, which may facilitate immune recognition and contribute to protective host responses.

Efficient recombinant expression of the vaccine protein was achieved in an *E. coli* expression system using the pET‐20b(+) vector, which is widely used for high‐yield recombinant protein production [22]. SDS‐PAGE analysis confirmed a distinct band at the expected molecular weight (~47.86 kDa), consistent with successful expression of the construct. TLR4 was selected as a target for computational analysis in this study due to its important role as an innate immune receptor involved in pathogen recognition and immune regulation [23, 24]. Molecular docking analysis demonstrated a favorable interaction between the designed MEV construct and the TLR4–MD2 complex, characterized by stable intermolecular contacts and favorable binding characteristics supporting the formation of a stable receptor–ligand complex. These findings indicate structural compatibility between the vaccine construct and the TLR4–MD2 complex and provide insight into the potential molecular interactions of the vaccine candidate. To further evaluate the stability and dynamic behavior of the predicted complex under physiological conditions, a 200‐ns MD simulation was performed. The RMSD, Rg, and PCA demonstrated that the vaccine‐bound TLR4–MD2 complex maintained a stable structural profile throughout the simulation period. Compared with the unbound system, the vaccine‐associated complex exhibited reduced conformational fluctuations and maintained a more organized structural state, suggesting that association of the vaccine construct may contribute to stabilization of the TLR4–MD2 complex. The LPS‐bound TLR4–MD2 complex was included as a reference system because LPS is a well‐characterized ligand known to interact with TLR4–MD2 and induce receptor‐associated conformational changes [25, 26]. As expected, the LPS‐bound system exhibited the highest degree of structural stability and compactness. Interestingly, the vaccine‐bound complex demonstrated a similar pattern of dynamic stabilization during the simulation, suggesting that the vaccine construct possesses structural characteristics that may influence the conformational behavior and stability of the TLR4–MD2 complex.

Previous studies have also explored HBHA‐containing vaccine constructs against infectious diseases. In particular, an HBHA‐containing MEV against *Leishmania infantum* was previously evaluated in BALB/c mice and showed immunogenic potential, supporting the use of HBHA as an immunomodulatory component in chimeric vaccine design [27]. HBHA has also been described as a protein immune adjuvant with TLR4‐associated immunostimulatory activity in previous studies. However, the present study did not experimentally isolate the contribution of HBHA from the other components of the vaccine construct; therefore, the observed immune responses cannot be attributed specifically to HBHA. The predicted interaction of the present HBHA‐containing construct with TLR4/MD2 is therefore consistent with previous computational investigations; however, docking and MD analyses provide structural insights and do not directly demonstrate TLR4 activation.

At the cellular level, cytokine profiling revealed a clear Th1‐skewed immune signature, which is considered important for controlling intracellular pathogens such as *Leishmania* spp. Elevated IL‐12 expression prior to challenge suggests effective activation of antigen‐presenting cells and induction of Th1‐associated immune responses [28]. Following challenge, IL‐12 expression declined relative to the prechallenge level, whereas IFN‐*γ* expression increased progressively. This cytokine profile may reflect the rapid production of IFN‐*γ* by previously primed antigen‐responsive T cells without requiring sustained high IL‐12 expression. The partial recovery of IL‐12 expression observed at Day 30 postchallenge may indicate continued antigenic stimulation contributing to the maintenance of a Th1‐associated immune environment. A transient increase in IL‐4 expression prior to challenge may reflect early immune activation; however, its specific cellular origin and functional significance require further investigation. Overall, the observed IL‐12/IFN‐*γ* expression pattern supports the establishment of a Th1‐oriented immune profile associated with enhanced cellular immune responses against intracellular pathogens such as *Leishmania* [3, 23]. It should also be noted that these findings are based on cytokine mRNA expression measured by quantitative RT‐PCR and therefore reflect transcriptional changes associated with vaccine‐induced immune responses. Although the present study focused on representative Th1/Th2 cytokines (IFN‐*γ*, IL‐12, and IL‐4), future studies incorporating additional cytokines, including TNF‐*α* and IL‐10, would provide a more comprehensive characterization of vaccine‐induced immune responses and the balance between protective and regulatory mechanisms.

Western blot analysis showed that sera from the vaccinated mice recognized both the recombinant multiepitope protein and distinct components of the *L. tropica* promastigote extract. Two prominent reactive bands of approximately 11 and 63 kDa were detected in the promastigote extract, whereas no corresponding reactivity was observed with sera from the PBS control group. These findings indicate that vaccination elicited antibodies that not only recognized the recombinant vaccine protein but also cross‐recognized native antigenic components of *L. tropica* promastigotes. Notably, the approximately 63‐kDa band may correspond to gp63, one of the parasite proteins represented among the antigenic sources incorporated into the vaccine construct, whereas the approximately 11‐kDa band may correspond to another parasite‐derived antigen included among the selected antigenic sources. However, because the identities of the reactive bands were not experimentally confirmed, these assignments remain tentative. Overall, the Western blot findings provide supportive evidence that the vaccine‐induced antibody response is capable of recognizing antigenically relevant components of the native *L. tropica* parasite extract.

Although the present findings demonstrate robust vaccine‐induced immunogenicity, future studies incorporating an additional irrelevant recombinant protein control with identical vector‐derived components but lacking the selected *Leishmania* epitopes would provide further confirmation of the epitope specificity of the elicited immune responses. Although recombinant protein expression in *E. coli* may be associated with residual LPS contamination [29, 30], the observed immune response is not consistent with LPS‐driven nonspecific activation. LPS typically induces broad innate immune stimulation without epitope‐specific antibody production [23, 24]. Furthermore, the cytokine profile exhibited a regulated and time‐dependent Th1‐skewed pattern, which is more consistent with antigen‐specific immune priming than endotoxin‐mediated inflammation. Therefore, although LPS contamination cannot be completely excluded, endotoxin removal and quantification should be considered in future studies.

The survival and safety observations further support the tolerability of the recombinant MEV. No mortality, behavioral changes, or apparent vaccine‐related adverse effects were observed during the observation period. Multiepitope and chimeric vaccine strategies have previously been investigated against different *Leishmania* species, including experimental evaluation in BALB/c mice and computational or in vitro assessment of recombinant vaccine constructs [31, 32]. These studies provide additional support for the continued investigation of epitope‐based vaccine approaches against leishmaniasis. Although survival alone cannot be interpreted as a direct measure of protective efficacy, it provides supportive information regarding vaccine tolerability during the observation period. Therefore, the current findings reinforce the suitability of the recombinant construct for further preclinical evaluation. The present study was primarily designed to characterize the immunogenicity and early immune activation induced by the MEV candidate following immunization. Although vaccinated animals were subsequently subjected to challenge, the experimental design was mainly focused on evaluating vaccine‐induced cellular and humoral immune responses rather than providing a comprehensive assessment of protective efficacy or long‐term immune durability. The observed immune responses demonstrate a robust immunogenic profile induced by the vaccine candidate. Future studies incorporating quantitative parasite‐burden assessment at defined postchallenge time points, together with protein‐level cytokine quantification and functional evaluation of antigen‐specific immune responses, will further clarify the protective potential of the vaccine candidate.

## 5. Conclusions

The present study provides experimental validation of a rationally designed MEV candidate against *L. tropica*. The construct was successfully expressed in a bacterial system and evaluated in a murine model, demonstrating its ability to induce coordinated cellular and humoral immune responses. These findings support the feasibility of epitope‐based vaccine strategies for generating targeted immune activation against CL and identify the designed construct as a promising candidate for further preclinical development. Although the immune responses elicited by the vaccine are consistent with those associated with protection, the present findings should be interpreted as evidence of immunogenicity rather than definitive protective efficacy. Future studies incorporating quantitative parasite‐burden assessment at defined postchallenge time points, together with longitudinal monitoring of footpad swelling, will further clarify the protective potential of the vaccine candidate.

**Nomenclature**
BALB/c: Bagg albino mouse strain
BCIP: 5‐bromo‐4‐chloro‐3‐indolyl phosphate
BL21 (DE3): *Escherichia coli* expression strain
CD4^+^
: cluster of differentiation 4 positive T cells
CD8^+^
: cluster of differentiation 8 positive T cells
cDNA: complementary DNA
CL: cutaneous leishmaniasis
CTL: cytotoxic T lymphocyte
DNA: deoxyribonucleic acid
EAAAK: glutamic acid–alanine–alanine–alanine–lysine linker
*E. coli*
: *Escherichia coli*
FBS: fetal bovine serum
FW: forward primer
GAPDH: glyceraldehyde‐3‐phosphate dehydrogenase
GPGPG: glycine–proline–glycine–proline–glycine linker
HBHA: heparin‐binding hemagglutinin
HTL: helper T lymphocyte
IFN‐*γ*
: interferon gamma
IL: interleukin
IPTG: isopropyl *β*‐D‐1‐thiogalactopyranoside
KMP11: kinetoplastid membrane protein 11
*L. major*
: *Leishmania major*
*L. tropica*
: *Leishmania tropica*
LBL: linear B lymphocyte epitope
MD: molecular dynamics
MEP: multiepitope protein
MEV: multiepitope vaccine
mRNA: messenger RNA
NBT: nitro‐blue tetrazolium
PADRE: pan‐DR epitope (AKFVAAWTLKAAA)
PBS: phosphate‐buffered saline
PCA: principal component analysis
PCR: polymerase chain reaction
pET‐20b(+): protein expression vector
qPCR: quantitative polymerase chain reaction
RE: reverse primer
rMEP: recombinant multiepitope protein
RNA: ribonucleic acid
RPMI: Roswell Park Memorial Institute medium
SDS‐PAGE: sodium dodecyl sulfate polyacrylamide gel electrophoresis
SPSS: Statistical Package for the Social Sciences
TBS‐T: Tris‐buffered saline with Tween‐20
Th1: T helper 1 cell response
Th2: T helper 2 cell response
TLR4: Toll‐like receptor 4

## Funding

No funding was received for this manuscript.

## Consent

The authors have nothing to report.

## Conflicts of Interest

The authors declare no conflicts of interest.

## Supporting information


**Supporting Information** Additional supporting information can be found online in the Supporting Information section. Material S1: This file presents the computational workflow used for the design of the multiepitope vaccine, including antigen selection, epitope prediction, immunoinformatic filtering, and structural evaluation. A summary of the bioinformatic tools, prediction servers, and selection criteria applied in this workflow is provided in Table S1. Table S1: This table summarizes the bioinformatic tools, prediction servers, and selection criteria used in the computational workflow, including the threshold values applied for antigenicity, allergenicity, toxicity assessment, and epitope screening. Material S2: This file presents the distribution of HLA alleles in the Syrian population used for population‐specific vaccine design, together with the complete list of predicted CTL and HTL epitopes prior to final screening. Material S3: This file provides the full amino acid sequence of the designed multiepitope vaccine construct, along with detailed annotation of its functional components, including the adjuvant, epitopes, linkers, and tags. Figure S1: This figure illustrates the molecular docking results between the multiepitope vaccine construct and the TLR4 receptor, highlighting the interaction pattern and stability of the docked complex generated using the ClusPro server.

## Data Availability

The data that support the findings of this study are available in the Supporting Information of this article.
