# Supplementary material for: In Silico Design and Immunogenicity Evaluation of a Multiepitope Vaccine Candidate Against Leishmania tropica in BALB/c Mice
Source: J Parasitol Res. 2026 Oct 19;2026:3953857. doi: 10.1155/japr/3953857 (PMC13680856; doi:10.1155/japr/3953857)
Supplement: Supplementary file 1 [file JAPR-2026-3953857-s001.docx]

Supplementary Material S1. Computational Workflow and Epitope Prediction Details

Description:
This supplementary material provides a detailed stepwise computational workflow employed in the rational design of the multi-epitope vaccine construct against *Leishmania major* and *Leishmania tropica*. It includes the complete pipeline of antigen selection, epitope prediction, and multi-layer immunoinformatic filtering to ensure high antigenic potential, safety, and population relevance. The workflow begins with the retrieval and screening of conserved and immunodominant Leishmania proteins, which were evaluated for antigenicity to identify suitable vaccine candidates. CTL, HTL, and linear B-cell epitopes were then predicted using immunoinformatics tools. Predicted epitopes underwent stringent multi-parameter filtering based on antigenicity, allergenicity, toxicity, and cytokine-inducing potential (IFN-γ and IL-4 responses). Furthermore, epitope selection was guided by population-specific HLA allele distribution, with emphasis on the Syrian population as well as murine MHC alleles to ensure both epidemiological relevance and experimental validation feasibility. The final vaccine construct was assembled using appropriate linkers and functional sequences, followed by structural modeling, physicochemical evaluation, molecular docking with the TLR4 receptor, and molecular dynamics simulation to assess structural stability and interaction dynamics under physiological conditions.

Supplementary Table S1. Bioinformatic tools and selection criteria used in this study

| **Analysis** | **Tool** | **Criteria / Threshold** |
| --- | --- | --- |
| Antigenicity prediction | VaxiJen 2.0 | ≥ 0.5 |
| CTL epitope prediction | NetMHCpan-4.1 | IC50 ≤ 50 nM, Rank < 0.5% |
| HTL epitope prediction | NetMHCIIpan-4.1 | IC50 < 500 nM, Rank < 5% |
| B-cell epitope prediction | Immune Epitope Database | Multi-tool consensus |
| Allergenicity | AllerTOP v2.0 | Non-allergen |
| Toxicity | ToxinPred | Non-toxic |
| IFN-γ induction | IFNepitope | Positive prediction |
| IL-4 induction | IL4Pred | Negative prediction |

Supplementary Material S2. HLA Allele Frequencies and Predicted Epitopes

Description:
This supplementary material presents the HLA allele distribution in the Syrian population used for population-specific vaccine design, along with the full list of predicted CTL and HTL epitopes prior to final screening.

S2.1 HLA Class I Alleles (Syrian Population)

- HLA-A: A*02, A*24, A*01, A*03
- HLA-B: B*35, B*51, B*44, B*52
- HLA-C: C*04, C*07, C*12, C*06
- Mouse alleles: H-2Kᵈ, H-2Dᵈ, H-2Lᵈ

**S2.2 HLA Class II Alleles (Syrian Population)**

- HLA-DRB1: DRB1*11, DRB1*04, DRB1*07
- HLA-DQB1: DQB1*03, DQB1*05
- Mouse alleles: H-2-IAᵘ, H-2-IEᵈ, H-2-IEᵏ

Supplementary Material S3. Vaccine Amino Acid Sequence and Construct Design

Description:
This supplementary material provides the full amino acid sequence of the designed multi-epitope vaccine construct and a detailed annotation of its functional regions, including adjuvant, linkers, tags, and selected CTL, HTL, and B-cell epitopes with their corresponding parent *Leishmania* proteins**.**

MAENSNIDDIKAPLLAALGAADLALATVNELITNLRERAEETRTDTRSRVEESRARLTKLQEDLPEQLTELREKFTAEELRKAAEGYLEAATSRYNELVERGEAALERLRSQQSFEEVSARAEGYVDQAVELTQEALGTVASQTRAVGERAAKLVGIELPKKAAPAKKAAPAKKAAPAKKAAAKKAPAKKAAAKKVTQKEAAAKAKFVAAWTLKAAAAAYRWVHRVPEFAAYYLAAVLEYLAAYAYGAAVQAFAAYSYSNRAAAYIAAYYMEDQRFALHEYGAEALERAGTKPLVRGVRNASHIHGPGPGELTLYANERTEGAGSGPGPGGDIIAQAQSGTGKTGGPGPGNPAEHTSYSNRAAAYGPGPGQRKYEAAIDLYKRALGPGPGRPSAKAAETAKPKEEKKFKVPQAHITEGFSNTKKEQQKAAQYPSKRVRRHHHHHH

Supplementary Figure S1. Molecular Docking Analysis

Description:

Molecular docking interaction between the multi-epitope vaccine construct and the TLR4 receptor. The docked complex was generated using the ClusPro server, and the selected model represents the lowest-energy conformation among the predicted docking models.

Supplementary Figure S1: Molecular docking interaction between the multi-epitope vaccine construct and the TLR4 receptor. The docked complex was generated using the ClusPro server.
